# Effect of Gong's Mobilization Versus Conventional Physiotherapy Among Type II Diabetic Patients With Adhesive Capsulitis

**DOI:** 10.7759/cureus.63325

**Published:** 2024-06-27

**Authors:** Swetha Kamani Raveendhra Babu, Vinodhkumar Ramalingam

**Affiliations:** 1 Physiotherapy, Saveetha College of Physiotherapy, Saveetha Institute of Medical and Technical Sciences, Saveetha University, Chennai, IND

**Keywords:** range of motion, diabetes mellitus, home remedies, mobilization, adhesive capsulitis

## Abstract

Background: Adhesive capsulitis (AC) causes a spontaneous onset of discomfort and a progressive loss of shoulder mobility among type II diabetic patients. These patients experience severe loss of shoulder function, which impairs their day-to-day tasks and requires immediate care. According to scientific evidence, Gong's mobilization has an immediate effect on improving shoulder mobility. Therefore, the objective of this study is to determine the effectiveness of Gong's mobilization along with conventional physiotherapy in order to reduce pain and enhance the range of motion (ROM).

Aim: This study aims to compare the effectiveness of Gong's mobilization and conventional physiotherapy for type II diabetic patients with AC.

Methods: This experimental study included 32 participants between the ages of 40 and 60 years who were diagnosed with shoulder AC which followed type II diabetes. The participants were randomly allocated into two groups: the Gong's mobilization group and the conventional physiotherapy group. The participants in both groups received the intervention for four weeks. Outcome measures used for assessment before and after treatment were the Shoulder Pain and Disability Index (SPADI) and ROM. Data was analyzed using paired and unpaired t-tests.

Results: This study's results suggested that there was a better improvement in the pre- and post-test mean values of SPADI and shoulder ROM (p < 0.001) in both groups following a four-week intervention. There was a statistically significant difference in the post-intervention results between the two groups (P <0.05), indicating that Gong's mobilization technique is more effective than conventional treatment.

Conclusion: The study findings showed reduced discomfort, and improved ROM and SPADI scores after the intervention. Both the treatments (Gong's mobilization and conventional approach) applied to AC for type II diabetes patients were beneficial. However, Gong's mobilization technique is more effective in treating AC in fewer treatment appointments compared with conventional therapy.

## Introduction

Adhesive capsulitis (AC) is a known musculoskeletal problem clinically diagnosed as a frozen shoulder (FS) [1]. The cause of AC is a self-limiting disorder, which is unknown. In specific with gender, men are less likely to be affected than women [2]. According to the literature, the estimated prevalence of type II diabetes individuals was 22.4% [3], in the age group of 40-60 years, and the length of diabetes linked to AC [4]. According to the International Diabetes Federation (IDF), there will be 642 million diabetic patients worldwide by 2040, accounting for 10.4% of the global population. Diabetes can harm the musculoskeletal system and lead to a host of other health issues, which increases the severity of the condition [5]. The main cause of AC in diabetic patients is elevated blood glucose levels which leads to shoulder inflammation and thickening of the capsule surrounding the shoulder joint. This inflammation occurs due to various mechanisms, including increased production of pro-inflammatory cytokines and activation of inflammatory pathways that promote scar tissue and adhesions within the joint capsule, causing pain and restricting movement. In the case of chronic inflammation, it may lead to excessive build-up of collagen and other extracellular substances, affecting tissue architecture. Over the bicipital groove and the humeral head, there is palpable discomfort. All directions of movement are restricted; flexion, abduction, and external rotation are the most frequent restrictions on the range of motion (ROM).

In many cases, the exact cause of AC is idiopathic. Certain risk factors and associations have been identified. The primary idiopathic stiff shoulder develops without any injury or other shoulder conditions but includes diabetes, Dupuytren contracture, thyroid-related issues, myocardial infarction, and Parkinson's disease. The secondary stiff shoulder is caused by intra-articular, capsular, extra-articular, or neurological conditions [6].

AC is divided into four stages, each of which has a unique clinical appearance. This phase lasts for more than three months, is marked by night-time shoulder discomfort, and is intact with glenohumeral mobility. In the freezing period, the glenohumeral joint becomes extremely painful and rigid during the three to nine months. The frozen phase, which occurs from nine to 14 months, is characterized by pain at the extremities of ROMs and widespread loss of mobility. The thawing phase is defined by little discomfort, an improvement in shoulder mobility, and persistent stiffness that lasts for 15 to 24 months [7]. This disease is characterized by a spontaneous onset of pain and restriction in shoulder mobility [8]. Besides, sleeping constantly on the same shoulder might increase the joint load, causing unpleasant sleep patterns and discomfort. To quantify both discomfort and pain, the Shoulder Pain and Disability Index (SPADI) was established which has high reliability and validity in assessing the shoulder impairments. In total, 13 items, five for pain and eight for disability. The visual analog scale (VAS) was used to assess pain in the first SPADI version [9]. The pain and disability elements are zero "no pain" and 10 "worst imaginable pain" respectively [10]. According to clinical findings, magnetic resonance imaging (MRI) shows thickening of the pericapsular tissues, loss of the axillary recess, glenohumeral gap thickening of the coracohumeral ligament, subcoracoid fat, and the severity of the problem [11]. This musculoskeletal condition requires a high-quality treatment such as physical therapy or in combination with steroids, which may reduce symptoms in three to four months. However, the most beneficial method for treating AC for diabetic patients is physical therapy, which involves joint mobilization, stretching, ROM and strengthening exercises, cold packs, ultrasounds, and Codman's pendulum movements [12]. Especially drugs like corticosteroids and oral medications are part of the conservative treatment that can be cured in two to three years [13].

Gong's mobilization technique is one of the mobilization techniques that helps the shoulder to maintain in a neutral position at the end of the ROM [14]. Pressures are supplied based on the patient's level of discomfort during mobilization procedures, which are carried out using Maitland grades [15]. Various approaches, such as distraction, are used to stretch soft tissues, increasing ROM by applying pressure, gliding, and twisting procedures. While performing anterior-posterior glide ask the patient to perform the limited motions as part of Gong's mobilization technique. This involves the shoulder in a dynamic position [16]. The conventional treatment is quite a common traditional treatment and takes additional time to give benefits to patients with AC. In conservative management, Codman's pendulum exercise became a traditional way to passively mobilize the glenohumeral joint without exposing newly healed or injured tissues [17]. On the other hand, Gong's mobilization is a more advanced method that provides an instant effect on relieving symptoms [18]. Furthermore, there are a limited number of studies that discuss diabetics in AC. Thus far, no study has been reported on the effectiveness of Gong's mobilization and conventional treatment for type II diabetic patients with AC in an experimental study.

## Materials and methods

**Figure 1:**
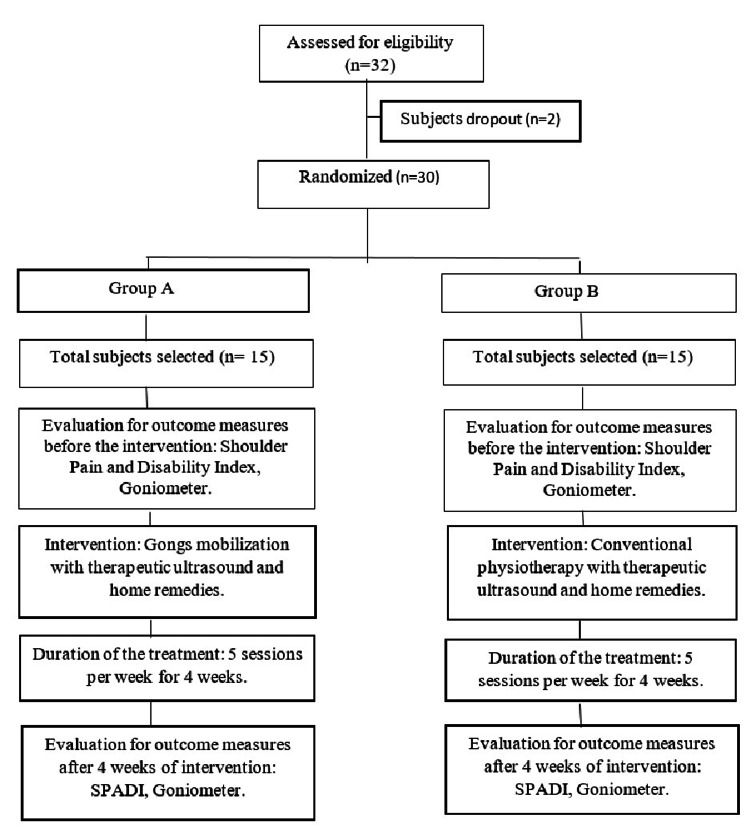
Flow chart for the methodology SPADI: Shoulder Pain and Disability Index

In this study, 32 participants were invited. Those who attended the OPD of physiotherapy, Saveetha Medical College and Hospital (SMCH) who had confirmed diagnosis of type II diabetes with AC were selected using a convenient sampling method. In this experimental study, both males and females were included between the age group of 40-60 years with decreased ROM (less than or equal to 120 degrees), pain, and mild to moderate levels of type II diabetics (below or equal to 200 mg/dL). The exclusion criteria were rheumatoid arthritis, malignancies, rotator cuff rupture, and severe levels of diabetes (above 400 mg/dL). Before beginning the treatment protocol, they underwent pre and post-tests, measured by SPADI and ROM. All participants were asked to sign their consent form. The instructions were clearly communicated to the participants regarding interventions and assessments. Thirty participants participated, whereas two participants were excluded due to a lack of follow-up. The participants were divided into two equal groups (consisting of n=15 participants in each group), that is Gong's mobilization group and conventional physiotherapy group, and each group received common home remedies and therapeutic ultrasounds. Mean and standard deviations of age, gender, pre- and post-test values were calculated. The total duration of the intervention was for four weeks as shown in Figure 1. The study was approved (01/030/2023/ISRB/SR/SCPT) by the Institutional Scientific Review Board, Saveetha College of Physiotherapy, Chennai.

Procedures

Gong's Mobilization

Applying the criteria of Gong et al. the patient is made to be in a lying position, while the affected arm is placed upwards to keep the patient's humerus upright, flexing the elbow 90 degrees, and abducting the shoulder 90 degrees [19]. The therapist later holds the patient's hand and applies anterior to posterior pressure to the humerus while keeping the elbow at a 90-degree angle. Then the therapist gently pressed on the articular capsule of the shoulder joint while raising the patient's torso. The articular capsule is gently pushed for 10 to 15 seconds and then released for five seconds. The therapist uses one hand to complete the shoulder's medial rotation while stabilizing the elbow. This technique is performed for five sessions per week for four weeks, 10-15 repetitions each time.

Conventional Physiotherapy

In Codman's pendulum exercise, the patient is made to bend over the front waist on a table supported by one arm. The painful arm is hung loosely. To improve on simple arm wiggles, weight is transferred from the front leg to the back leg. The arm is swung forward, backward, and sideways. It is repeated for 10-15 repetitions for five sessions per week for four weeks.

Therapies

Both groups of patients received the therapeutic ultrasound and home remedies to alleviate discomfort.

Therapeutic Ultrasound

The patient is made to be in a comfortable position and therapeutic ultrasound is given to the participants at a frequency of 3 MHz using parametric parameters. For eight minutes of treatment, a 5 cm^2^ transducer is utilized in continuous mode with 1.5 W/cm^2^ intensity. After putting the coupling medium (aqua sonic gel) in the transducer, the treatment head is slowly moved in a circular, overlapping pattern across the front, top, and back of the shoulder areas. The therapeutic ultrasound is given five times a week for four weeks.

Home Remedies

For the wand exercise, the wand is held behind the back; the palms should face up while the patient is standing on their feet. The wand is gently moved forward and backward until the anterior shoulder area feels stretched. This exercise is held for five to 10 seconds. Beginners should start with a few repetitions and gradually increase the volume of exercise. Repeat this exercise for 10 repetitions for five sessions per week for four weeks.

For the finger ladder exercise, the shoulder distance has to be kept around two feet from the walls. Next, the arm is raised to shoulder height and the fingertips are gently "walked" up the wall. The fingers are walked downward after that. It is repeated for 10 repetitions for five sessions per week for four weeks.

For the shoulder towel strengthening exercise, a tightly rolled-up towel is held by both hands. Using both hands, the towel is slowly pulled towards the ceiling. The bottom hand is slowly moved up the back by stretching the shoulder of the opposing arm. It has to be held for approximately 30 seconds. It is repeated for 10 repetitions for five sessions per week for four weeks.

For rope and pulley exercise, the patient's chair faces the wall which is secured with a rope and pulley. The rope handles are to be grabbed with both hands, palms facing out. Pulling the injured arm with the normal arm causes it to gently extend upward and downward. Next, the arm is to be dropped to the floor. This exercise is recommended to improve shoulder flexion ROM. For four weeks, 10 repetitions are to be performed over five sessions per week.

For the anterior shoulder stretching exercise, the body is to be rotated body sideways until the anterior portion of the shoulder stretches while holding a wall or other stable object at shoulder height. For four weeks, 10 repetitions are to be performed over five sessions per week.

Statistical analysis

The collected data was analyzed and tabulated using descriptive and inferential statistics. The data was tested for statistical significance. The groups passed the normality test checked by Shapiro-Wilk [20]. All parameters were analyzed using the mean and standard deviation. The paired t-test was used to analyze the data among the groups. The unpaired "t" test was used to compare the pre-test and post-test scores of two different groups. The unpaired "t" test was used to determine significant differences between the two groups, with a significance level of p < 0.05.

## Results

In this study, the participants were selected between the age group of 40-60 years. The mean age value of group A is 49.47 and group B is 50.13. Among the 30 participants, there were 19 females and 11 males. The mean age group and gender of the participants with percentage (Table 1).

**Table 1:** Age and gender of participants with the percentage

Age		Groups		Total
		Gong's mobilization group	Conventional physiotherapy group	
40-45		6 (20%)	5 (16.6%)	11 (36.7%)
46-50		2 (6.6%)	3 (10%)	5 (16.7%)
51-55		3 (10%)	1 (3.3%)	4 (13.3%)
56-60		4 (13.3%)	6 (20%)	10 (33.3%)
Gender	Male	5 (16.7%)	10 (33.3%)	15 (50%)
	Female	6 (20%)	9 (30%)	15 (50%)

When compared to the pre-assessment, the post-assessment shows a significant improvement in ROM and SPADI in four weeks of duration. In both Gong's mobilization group and conventional group, there was a statistically significant difference between the pre-test and post-test scores of SPADI. The statistical mean value of SPADI for pre-intervention in Gong's mobilization group is mean (71.1400), SD (3.86); post-intervention mean (40.5000), SD (5.89); t(df)=14; t-value 18.313; and the p-value is <0.001. For the conventional group, the pre-intervention mean value is (69.580), SD (5.64); the post-intervention mean value is (41.733), SD (4.90); t(df)=14; t-value 22.420, and the p-value is <0.001 (Table 2).

**Table 2:** Pre- and post-test values of SPADI for Gong's mobilization and conventional physiotherapy SPADI: Shoulder Pain and Disability Index

		Gong's mobilization				Conventional physiotherapy			
Outcome measure	Test	Mean	SD	t-value	p-value	Mean	SD	t -value	p-value
SPADI	Pre-test	71.140	3.86	18.313	<0.001	69.580	5.64	22.420	<0.001
	Post-test	40.500	5.89			41.733	4.90		

**Figure 2:**
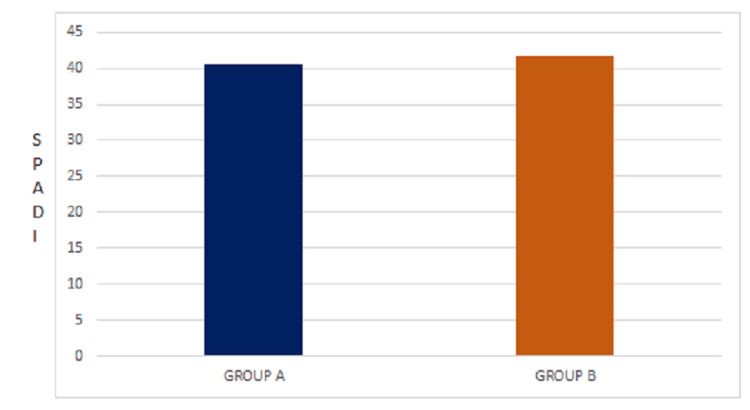
Mean values of Gong's mobilization and conventional physiotherapy for SPADI SPADI: Shoulder Pain and Disability Index

The mean values of both Gong's mobilization and the conventional group are 40.50±5.89 and 41.73±4.90, t(df)=28, t-value-.623, and the p-value is .507 (Table 3, Figure 2).

**Table 3:** Mean values of Gong's mobilization group and conventional group for SPADI SPADI: Shoulder Pain and Disability Index

Outcome measure	Groups	Mean	SD	t-value	p-value
SPADI	Gong's mobilization	40.5000	5.89310	-.623	.507
	Conventional physiotherapy	41.7333	4.90956		

Both groups have shown improvement in post-intervention for SPADI. There is also a difference in the ROM of the shoulder. In the Gong's mobilization group, the pre-intervention mean value of motions is flexion = (76.26) SD (12.25), extension = (20.13) SD (3.58), abduction = (77.00) SD (10.48), internal rotation = (27.33) SD (4.67), and external rotation = (26.20) SD (3.98). The post-intervention mean values are flexion = (141.66) SD (8.44), t(df)=14, t-value -46.489, the p-value is <0.001, extension (35.00) SD (5.34), t(df)=14, t-value -12.613, the p-value is <0.001, abduction = (143.86) SD (7.74), t(df)=14, t-value -23.421, the p-value is <0.001, internal rotation = (51.66) SD (8.12), t(df)=14, t-value -10.875, p-value is <0.001 and external rotation = (46.60) SD (8.31), t(df)=14, t-value -10.390, p-value is <0.001. In the conventional group, the pre-intervention mean values are flexion= (68.80) SD (13.62), extension= (19.20), SD (2.93), abduction = (70.66) SD (9.23), internal rotation = (27.13) SD (4.5), external rotation= (26.46) SD (4.18). The post-intervention mean values of flexion = (132.40) SD (7.99), t(df)=14, t-value -21.091, the p-value is <0.001, extension = (29.73) SD (2.96), t(df)=14, t-value -10.540, the p-value is <0.001, abduction= (139.53) SD (12.26), t(df)=14, t-value -19.842, the p-value is <0.001, internal rotation = (46.53) SD (6.65), t(df)=14, t-value -10.659, the p-value is <0.001, external rotation = (42.93) SD (5.73), t(df)=14, t-value -11.905, and the p-value is <0.001 (Table 4).

**Table 4:** Pre- and post-values of ROM for Gong's mobilization and conventional physiotherapy ROM: range of motion

		Gong's mobilization				Conventional physiotherapy			
Motions	Test	Mean	SD	t-value	p-value	Mean	SD	t-value	p-value
Flexion	Pre-test	76.26	12.25	-46.489	<0.001	68.80	13.62	-21.091	<0.001
	Post-test	141.66	8.44			132.40	7.99		
Extension	Pre-test	20.13	3.58	-12.613	<0.001	19.20	2.93	-10.540	<0.001
	Post-test	35.00	5.34			29.73	2.96		
Abduction	Pre-test	77.00	10.48	-23.421	<0.001	70.66	9.23	-19.842	<0.001
	Post-test	143.86	7.74			139.53	12.26		
Internal rotation	Pre-test	27.33	4.67	-10.875	<0.001	27.13	4.54	-10.659	<0.001
	Post-test	51.66	8.12			46.53	6.65		
External rotation	Pre-test	26.20	3.98	-10.390	<0.001	26.46	4.18	-11.905	<0.001
	Post-test	46.60	8.31			42.93	5.73		

**Figure 3:**
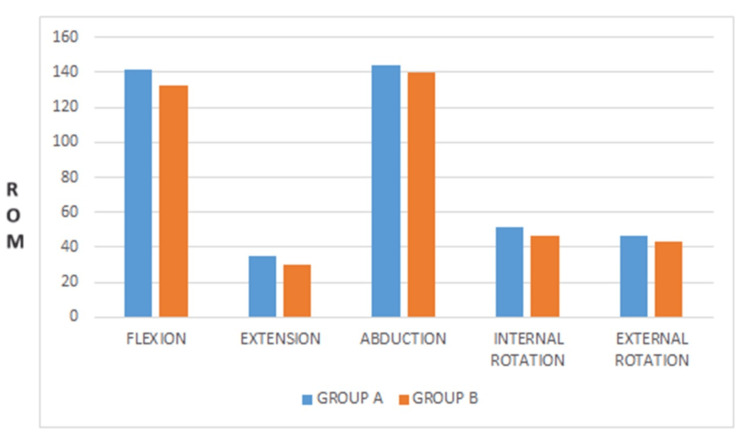
Mean values of ROM for Gong's mobilization and conventional physiotherapy ROM: range of motion

The mean values of the ROM for both the groups are flexion (141.66±8.44) (132.40±7.99), t(df)=28, t-value 3.086, p-value is .913, extension (35.00±5.34) (29.73±2.96), t(df)=28, t-value 3.338, the p-value is .020, abduction (143.86±7.74) (139 ±12.26), t(df)=28, t-value 1.157, the p-value is .029, internal rotation (51.66±8.12) (46.53±6.65), t(df)=28, t-value 1.894, the p-value is .860, external rotation (46.6± 8.31) (42.93± 5.71), t(df)=28, t-value 1.406, the p-value is 0.084 (Table 5, Figure 3).

**Table 5:** Mean values of ROM for Gong's mobilization and conventional physiotherapy ROM: range of motion

Motions	Groups	Mean	SD	t-value	p-value
Flexion	Gong's mobilization	141.66	8.44	3.086	.913
	Conventional physiotherapy	132.40	7.99		
Extension	Gong's mobilization	35.00	5.34	3.338	.020
	Conventional physiotherapy	29.73	2.96		
Abduction	Gong's mobilization	143.86	7.74	1.157	.029
	Conventional physiotherapy	139.53	12.26		
Internal rotation	Gong's mobilization	51.66	8.12	1.894	.860
	Conventional physiotherapy	46.53	6.65		
External rotation	Gong's mobilization	46.6	8.31	1.406	0.084
	Conventional physiotherapy	42.93	5.71		

From the results, it is found that the mean improvements in both groups were found to be statistically significant in four weeks of duration. According to the above-mentioned results, Gong's mobilization group and conventional group show greater improvement. Hence, the result of this study conveys that in aspects of pain, disability, and ROM, Gong's mobilization groups show a substantial improvement in treating type II diabetic patients with AC.

## Discussion

AC is characterized by early pain followed by progressive restriction of active and passive glenohumeral joint ROM. This condition primarily affects elderly people. Various methods have been explored to overcome this issue. This study determines whether Gong's mobilization and conventional physiotherapy, combined with therapeutic ultrasound and home remedies, can help type II diabetic patients with AC by improving ROM, decreasing pain and discomfort, and improving quality of life. There is a difference in pre- and post-intervention values for the outcome measures evaluated by SPADI and ROM.

Gong W, in 2020 concluded that the post group's flexion and abduction ranges of motion were significantly greater [21]. The pathology of AC causes stiffness in the joint capsule and other periarticular tissues, Gong's mobilization allows the tensed soft tissues to be stretched. The transitional movement facilitates the restoration of the shoulder's natural physiological motions which has positive effects and increases ROM [21]. As in the case of Gong W, this study also mentions that post-group values for extension and abduction ROM were significantly greater in four weeks of duration [21]. This indicates a positive result in both the Gong's mobilization group and the conventional group. It increases functional ability and shoulder motions, decreases pain, and improves quality of life.

Sung et al. in 2022 concluded that combining ultrasound deep heat therapy as a co-intervention with additional physical modalities can effectively alleviate pain in patients with AC [22]. In this study's findings, conventional treatment which includes ultrasound and exercise gives better relief by decreasing the pain over the shoulder joint and increasing shoulder mobility [22]. Normally, AC patients experience pain and discomfort, functional limitations, and muscle weakness. According to Sung et al., ultrasound and exercise will relieve pain [22]. In our view, if conventional treatment alone is given to the patients, it may take a longer duration to cure, but conventional combined with other mobilization techniques will give faster results.

According to Nakandala et al. in 2021, there is empirical evidence that certain physical therapy approaches and modalities are strongly suggested for pain reduction, increased ROM, and functional ability in patients with AC, while others are moderately or slightly advised [23]. However, the superiority of one therapy modality over another is unclear. The lack of scientific accuracy exhibited in the majority of the examined studies emphasizes the critical need for properly conducted, adequately randomized controlled trials with enough follow-up to determine the optimal combination of treatments [23]. This study validates our study, that mobilization and conventional physiotherapy give the best results. Giving only conventional treatment takes more time to cure the patients with AC, but it reduces the pain and improves shoulder motions.

Sghir et al. (2020) concluded that both diabetic acute coronary syndrome (ACS) and idiopathic ACS were equal in terms of epidemiologic characteristics (p < 0.05) [24]. Compared to diabetic ACS, idiopathic ACS was significantly more prevalent in women (p = 0.009). The two groups' VAS scores were initially statistically equal (p > 0.05). Abduction and external rotation mobility did not significantly differ between the groups at the beginning of the study (p > 0.05). At baseline, there was statistical equality in the HBB reach levels in both groups (p > 0.05). The first revised evaluations from constant were statistically equal. The idiopathic ACS patients reported significantly less pain than the DM patients when comparing the VAS pain levels recorded at baseline and following the last therapeutic session in both groups (p 0.001). Therefore, this study recommends employing rehabilitation as an effective therapy in practice for ACS that, according to VAS and goniometer results, dramatically lowers pain and enhances ROM [24]. Pain intensity and functional capacity will typically differ between diabetes and non-diabetic people. Also, pre- and post-test values will vary for diabetic patients according to their blood glucose levels. So, in this study, type II diabetic patients were included below the level of 200 mg/dL. High glucose levels, more than 400 mg/dL, were excluded from this study due to the high-risk factor involved. This study also shows significant improvement (p <0.005).

In 2022, Kariya et al. concluded that the effectiveness of Gong's mobilization in this frozen shoulder case before and after intervention, as well as the patient's ROM, manual muscle testing, and pain assessment before and after intervention using the numerical pain rating scale, were investigated, allowing the therapist to determine the efficiency of this mobilization [25]. Many studies recommend the use of various mobilization techniques and therapy protocols to stop disease progression and alleviate symptoms. The purpose of this case report is to raise awareness of this type of mobilization and to highlight it as a new and recent advancement in the treatment of frozen shoulders. It also tries to provide enough treatment to alleviate the aforementioned ailment and improve mobility [25].

In 2020, Ramteke et al. concluded that combining Gong's mobilization with conventional therapy is more effective for treating frozen shoulders than using conventional therapy alone in improving pain and ROM. However, Gong's mobilization led to a greater increase in ROM and a decrease in discomfort. The study suggests that combining Gong's mobilization with conventional therapy can effectively treat patients with frozen shoulders [26].

Limitations and recommendations

People who have type II diabetics with AC were included in this study. It is advised that upcoming research should focus on patients who have type I diabetics and specific stages of AC. The influence of the condition on occupational health should also be taken into consideration when selecting participants, and additional measurement instruments can be used to rule it out. Participants can be taken based on other specific metabolic disorders rather than diabetics.

## Conclusions

This study conveys to clinical therapists to incorporate Gong's mobilization as a part of physiotherapy management while handling patients with adhesive capsulitis (AC). This study found that Gong's mobilization and conventional physiotherapy seem to be effective in treating type II diabetic patients with AC. The patients in Gong's mobilization group showed greater improvement than the conventional group. Hence this study concluded that Gong's mobilization technique was more effective when compared with conventional physiotherapy in improving the range of motion and functional activity, and reducing shoulder pain and shoulder discomfort in type II diabetic patients with AC.
